# Neurofilament Proteins as Body Fluid Biomarkers of Neurodegeneration in Multiple Sclerosis

**DOI:** 10.1155/2011/315406

**Published:** 2011-01-23

**Authors:** Melissa M. Gresle, Helmut Butzkueven, Gerry Shaw

**Affiliations:** ^1^Department of Medicine, University of Melbourne, Parkville, Victoria 3010, Australia; ^2^Florey Neuroscience Institutes, Howard Florey Institute, Parkville, Victoria 3010, Australia; ^3^Department of Neuroscience, McKnight Brain Institute, University of Florida College of Medicine, Gainesville, FL 32610, USA; ^4^EnCor Biotechnology Inc., Suite 40, 4949 SW 41st Boulevard, Gainesville, FL 32608, USA

## Abstract

Biomarkers of axonal degeneration have the potential to improve our capacity to predict and monitor neurological outcome in multiple sclerosis (MS) patients. Neurofilament proteins, one of the major proteins expressed within neurons and axons, have been detected in cerebrospinal fluid and blood samples from MS patients and are now being actively investigated for their utility as prognostic indicators of disease progression in MS. In this paper, we summarize the current literature on neurofilament structure, assembly, and degeneration and discuss their potential utility as biomarkers for monitoring neurological decline in MS. We also discuss the need to further develop sensitive methods for assaying neurofilaments in blood to improve clinical applicability.

## 1. Introduction

Multiple Sclerosis (MS) is a chronic, debilitating neurological disease with an unknown aetiology. The pathology of this disease is complex and heterogeneous but is typically characterized by the presence of multifocal demyelinated plaques, inflammation, and axonal injury [1]. The functional consequences of this pathology can include visual disturbances, fatigue, depression, weakness, numbness, and cognitive impairment. The earliest symptoms typically begin in young adulthood [2], a time when the diagnosis of an unpredictable, chronic neurological disease with significant and often progressive disability is particularly devastating and unexpected.

Increasingly, axonal injury is recognized as the main pathological correlate of progressive neurological disability in MS [3, 4]. Historically, this axonal damage was thought to be restricted to chronically demyelinated lesions, caused by trophic factor deprivation [5] or maladaptive responses in chronically demyelinated axons [6]. Several histological and imaging studies have now demonstrated, however, that axonal damage may also occur in association with inflammation in acute grey and white matter lesions, and also more diffusely in normal-appearing white matter [7–9]. 

 At present, surrogate markers for axonal damage are not routinely used to monitor disease activity in MS patients. The most commonly used diagnostic and monitoring tool for MS is magnetic resonance imaging, utilizing T_2_-weigheted imaging and Gadolinium- (Gd-) enhanced T_1_-weighted imaging [10]. These measures, however, lack pathological specificity, which is likely to contribute to the poor association between conventional MRI measures and disability in MS patients [11, 12]. To address this need, several studies have now focussed on the detection of neuronal/axonal proteins in CSF or blood, as biomarkers of axonal degeneration. These studies are based on the concept that degenerating axons release their contents into the surrounding extracellular space, and that some of these axonal components might be abundant and stable enough to be detectable with appropriate assays. The detection of such components would provide a convenient means to assess the presence and degree of axonal degeneration in MS, and this information could be useful for predicting and monitoring the progression of the disease, and for assessing the efficacy of therapeutic strategies that are aimed at preventing axonal loss.

## 2. Introduction to Neurofilament Proteins

**Figure 1:**
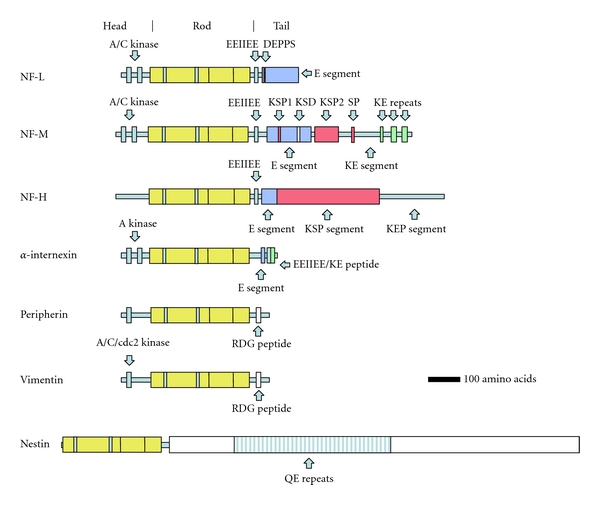
Diagram of the subunit proteins of neurofilaments. NF-L, NF-M, NF-H, and *α*-internexin can be regarded as the major subunits of adult NFs though NFs may also contain peripherin, vimentin, and nestin in certain locations, developmental stages, and possibly damage or disease states. Phosphorylation sites for protein kinase A (A kinase), protein kinase C (C kinase), and cdc2 kinases (cdc2 kinase) have been characterized in the globular “head” regions of certain of these molecules as indicated. The regions indicated by KSP, SP, KSD, and DEPPS are known serine phosphorylation sites in the “tail” regions. EEIIEE, KE repeats, Tail a, E segment, KEP segment, KE segment, RGD, and QE repeats each refer to specific kinds of sequence motif. For further details, see Shaw 1998 [13].

Neurofilaments (NFs) are the major structural proteins of neurons. They are most abundant in larger neurons and are heavily concentrated in axons, in particular long projection axons. The subunits of NFs belong to the intermediate (IF) family of proteins, which are characterized by a structurally conserved *α*-helical coiled-coil “rod” region which forms the backbone of the filament, with variable N and C terminal extensions (Figure 1). The IF subunits of vertebrates are divided into five classes based on protein characteristics, expression pattern, and intron placement. IF subunits Class I and II include the epithelial keratins; the class III IFs include vimentin, desmin, and glial fibrillary acidic protein (GFAP), while the major neurofilament subunits NF-Light (NF-L), NF-medium (NF-M), NF-Heavy (NF-H), *α*-internexin, and nestin form IF class IV. Class V IFs are the lamin proteins of the nuclear matrix. The expression profile of Class IV proteins is limited to the nervous system, with the exception of nestin, which may be found in stem cells throughout the body. All 5 Class IV genes share a distinct intron pattern from that seen in other IF genes, indicating a close evolutionary relationship. The class IV subunits NF-H and NF-M have unusually long and complex C-terminal “tail” regions which are responsible for the wispy spacers seen by electron microscopy and the wider spacing of NFs compared to other IFs. NF-L, NF-M, NF-H, and *α*-internexin are all abundant proteins of the nervous systems of adult mammals, while nestin is expressed early in development and is normally downregulated in the adult. One class III IF protein, peripherin, is found copolymerized with NF-L, NF-M, NF-H, and *α*-internexin in the NF of some neurons in significant amounts, particularly in the peripheral nervous system. Finally, a few apparently unusual neurons in the adult express another Class III protein, vimentin. Neuroblasts express this protein, but it is generally downregulated as development proceeds. However, in many damage and disease states, cells will re-express proteins which were downregulated developmentally, so that expression of any of the 7 subunits shown in Figure 1 could be associated with specific damage or disease states. The various proteins which may be included in NFs are known to be phosphorylated, glycosylated, and modified on many sites and contain many interesting protein sequence motifs, details of which are discussed in previous publications [13, 14].

It is noteworthy that for a typical large projection neuron, the volume of the axon may exceed that of the cell body by a factor of a thousand or more and that NFs can occupy more than 90% of the axonal cross section. The function of NFs appears to be to provide axons with mechanical strength and to control axonal volume. Apparently, in order to meet these requirements, NF subunits have a very long half-life and are resistant to endogenous proteases. It is therefore reasonable to assume that large amounts of NFs, their subunits, breakdown products, and associated proteins would be released following axonal loss in MS and in other damage and disease states, and that their abundance and stability might make them relatively easy to detect. There has therefore been much interest in the use of these proteins as potential biomarkers of damage, disease, and progression in a variety of neurological states, including MS [15]. 

## 3. Neurofilaments As Biomarkers of Axonal Degeneration in MS

Several studies have now demonstrated the presence of NF peptides in the cerebrospinal fluid (CSF) of MS patients (summarized in Table 1). Most commonly, CSF levels of these proteins have been assessed using enzyme-linked immunosorbent assays (ELISAs) [16–20]; however, Western blotting and dot blotting have also been utilized on occasion [21–23]. 

### 3.1. NF-L

The potential use of the NF protein subunits as surrogate markers of axonal degeneration in MS was first explored by Lycke et al. [16], who developed an NF-L ELISA in house using an affinity purified chicken NF-L antibody. In this study, CSF levels of NF-L protein were measured in 60 patients with clinically definite relapsing-remitting MS (RR-MS). The CSF samples were collected from patients on initiation of the trial, and then 2 years later. It was demonstrated that CSF NF-L levels were increased on at least one occasion in 78% of cases, and that these levels were moderately associated with disability. Levels of NF-L were also found to be higher in patients who had suffered a relapse within 3 months of sampling, indicating a temporal correlation between acute inflammatory activity and neurodegeneration in this disease. An interesting observation to arise from this study was that sequential CSF NF-L samples were not persistently elevated in all patients, highlighting the dynamic nature of MS-associated axonal degeneration. 

Increases in intrathecal NF-L were subsequently confirmed by several groups, for both RR-MS and progressive MS cases [17, 23–25]. In accordance with original observations made byLyke et al.[16], a small prospective study of 13 RR-MS patients with recent relapse confirmed that NF-L levels peak during acute relapse and decline within 3 months [17]. In this same study, CSF NF-L levels were also assessed in a larger group of 66 patients with clinically definite MS for associations with disability as assessed by expanded disability status scale (EDSS) and neurologic symptoms. The level of NF-L in CSF was not found to be associated with neurological disability outcome measures. 

In a more recent set of retrospective studies, NF-L levels were measured from CSF samples collected at diagnostic lumbar puncture in 99 patients with clinically definite MS, to evaluate whether NF-L levels at diagnosis could be used to predict more rapidly progressing disease. Of these, 94 patients had comprehensive clinical data available to conduct association studies between CSF NF-L levels at diagnosis, and disease severity at 5 years [26] and 14 years [27]. It was found that elevated NF-L levels were associated with a 3-fold increase in the risk of developing severe MS, as estimated by bivariate and multivariate logistic regression analysis, particularly among cases with RR-MS and cases with a recent relapse. Further, approximately 60% of patients with high CSF NF-L levels (>386 ng/mL) converted from RR-MS to secondary progressive MS (SP-MS) within the 14-year followup period compared to 30% of patients with moderate or low levels (<386 ng/mL). These studies suggest that high CSF NF-L levels, assessed in early MS, are potentially predictive of more rapid disease progression over time. High NF-L levels in early MS may also be useful for predicting conversion to progressive disease. In accordance with these studies, it has also been reported that CSF NF-L levels are higher, on average, in clinically isolated syndrome (CIS) cases that convert to RR-MS within 3 years compared to nonconverters [18]. Collectively, this recent work supports the use of NF-L CSF levels as a prognostic indicator of disease course in MS patients.

### 3.2. NF-H

The high-molecular-weight NF subunit, NF-H, has also been a focus of biomarker studies. Compared with assessments of CSF NF-L levels, however, fewer studies have been conducted to evaluate NF-H as a biomarker of neurodegeneration in MS. The axonal form of NF-H is heavily phosphorylated [21], is resistant to proteolysis [29], and is very immunogenic, which allows it to be sensitively detected using appropriate immunological assays [30]. The phosphorylated region of this axonal form of NF-H (here referred to as pNF-H) is also very unusual, comprising ~50 tandem repeat lysine-serine-proline (KSP) containing peptides, the serine of each being a phosphorylation site [13, 31]. These unusual properties make pNF-H an ideal target for immunological detection, since it is stable upon release from neurons and can be captured and detected with exceptionally high avidity due to its exotic multiepitope nature. Since pNF-H is only found in axons, its detection in CSF, blood or other bodily fluids points unambiguously to release of this protein from axons.

 The first ELISA method described for NF-H made use of the commercial SMI35 monoclonal antibody in the capture role [32]. SMI35, available from Covance (Princeton, NJ) is specific for the axonal, heavily phosphorylated form of NF-H, namely pNF-H. This ELISA was subsequently utilized in a 3-year followup study conducted to evaluate CSF levels of pNF-H in RR-MS and progressive MS cases. In contrast to observations for NF-L, the median pNF-H levels were found to be highest for patients with progressive disease [19]. There was also some evidence to support an association between pNF-H level and EDSS at followup, and also a potential propensity for patients with high pNF-H levels at baseline to exhibit progression in disease EDSS at followup. These analyses did not, however, reach statistical significance, most likely due to the limited sample size. In support of these observations, CSF pNF-H levels were shown to be weakly associated with EDSS (*R* = 0.253, *P* < .009) in a larger cohort of patients comprising CIS (*n* = 38), RR-MS (*n* = 42), SP-MS (*n* = 28), and primary progressive MS (PP-MS, *n* = 6) cases [18]. In this study, the average CSF pNF-H levels were higher in patients with all subtypes of MS relative to samples taken from controls. Interestingly, CSF pNF-H levels were, on average, 1.5-fold higher for SP-MS and PP-MS cases relative to RR-MS cases. Further, in contrast to observations for NF-L, levels of CSF pNF-H in CIS cases did not predict conversion to clinically definite MS. These studies suggest the pNF-H may be more useful as a measure of ongoing neurodegenerative activity in MS patients, which would make this protein a potential candidate for use as a surrogate marker for assessment of treatments aimed at reducing axonal injury. A retrospective study of 30 patients has already been conducted to determine whether plasma pNF-H levels could be used to measure responsiveness to interferon-*β* treatment. Although it was found that plasma pNF-H levels tended to be higher in patients who did not respond well to Interferon-*β* treatment, this did not reach statistical significance, possibly due to the small sample size. Hence, additional studies are required to assess the utility of blood or CSF pNF-H levels as an indicator of disease activity and, potentially, therapeutic efficacy.

### 3.3. NF-M and *α*-Internexin

The other major neurofilament subunits of the mature nervous system, NF-M and *α*-internexin, are presumably, like NF-L and NF-H, released during axonal degradation. Some evidence suggests that *α*-internexin is a particularly unstable protein, difficult to isolate biochemically because it is readily degraded [33], and therefore the intact form of this protein is unlikely to be a suitable biomarker. On the other hand, the NF-M appears to be intermediate in resistance to proteases compared to NF-L and NF-H but has not, to date, been studied as a potential biomarker [34].

## 4. Axonal Injury Biomarker Panels Utilising Neurofilament Proteins

The use of multiple axonal injury biomarkers in combination panels in CSF has been explored as a method to gain additional power to explore disease prognosis and activity. The premise behind these studies is that individual axonal biomarkers may only reflect particular aspects of disease activity or may be released at different stages in the degenerative process and are therefore likely to be less informative in isolation than in combination. 

Studies by Brettschneider et al. [28] measured CSF levels of the axonal cytoskeletal proteins microtubule-associated protein tau and pNF-H in the same MS patients to ascertain whether this could improve the sensitivity of predicting disability progression relative to conventional MRI methods. Interestingly, when utilized alone, changes in CSF pNF-H or tau levels did not increase the sensitivity for predicting conversion to clinically definite MS in patients with CIS at 48-month followup. However, by testing CSF for either tau or pNF-H, it was possible to improve the sensitivity and specificity of these biomarkers relative to MRI. Interestingly, higher CSF levels of pNF-H, but not tau, were found to be associated with higher EDSS in both CIS and RR-MS, which may indicate that NF-H proteins are more sensitive indicators of axonal damage that is associated with physically disabling symptoms of MS. 

Teunissen et al. [18] have also evaluated the utility of combinations of axonal biomarkers for monitoring disease activity in MS patients. In their study, levels of neuron-specific amino acid n-acetyl aspartate (NAA), NF-L, pNF-H, and tau were measured in the same CSF samples and were then assessed for associations with disease subtype, disability, and MRI outcome measures. Each of these proteins showed specific patterns of change over the course of the disease, with the highest average NF-L levels observed in RR-MS patients. Conversely, average NAA and pNF-H levels were highest in patients with progressive disease. Levels of tau were not significantly changed between the groups. It was proposed that these differences could reflect variability in the dynamics of release for these axonal proteins during degenerative processes. However, both of these observations need validation in independent cohorts.

## 5. Neurofilament Protein Subunits As Blood Biomarkers of Axonal Degeneration in MS

Despite these promising observations, the clinical utility of CSF biomarkers is limited by the lack of acceptability, invasiveness, and risks posed by repeated lumbar puncture. Hence, blood biomarkers of axonal degeneration could provide a major advance in the paraclinical monitoring of disease activity in MS. The development of methods for measuring biomarkers in serum is, however, complicated by the potential influence of the blood brain barrier on the dynamics of release of these proteins into blood and the much higher protein concentration and complexity of blood as compared to CSF. In addition, proteins released into blood may be subjected to proteolysis and may bind to blood components that are actively cleared from the blood. However, blood brain barrier compromise appears to be an early feature of the MS disease process [35], and presumably, potential axonal biomarker proteins released into the extracellular space in lesions would have ready access into blood from sites of active MS pathology. Such proteins could then be detected with assays of sufficient affinity and avidity to work effectively in the concentrated and complex protein context of blood, plasma, and serum.

Relatively few studies have evaluated blood biomarkers as potential measures of neurodegeneration in MS. Thus far, a suitable method for detecting NF-L in blood has not been identified. The pNF-H protein, however, has been detected in blood samples, using two independently developed ELISA methods. The SMI35-based pNF-H ELISA was used to show that in a small group of 30 RR-MS patients, median plasma pNF-H levels were elevated relative to healthy controls [20]. This same method was used to demonstrate that in patients with acute optic neuritis, elevated plasma NF-H levels were associated with poor recovery of visual acuity and were also inversely correlated with visual acuity at presentation [36]. 

One of the present authors (G. Shaw) has also published a pNF-H assay which used an affinity purified chicken polyclonal antibody in that capture role (C*α*-pNF-H ELISA) [37], which has become commercially available from EnCor Biotechnology Inc. (Gainesville, FL), from BioVendor (Modrice, Czech Republic) and from Millipore (Billerica, MA). This assay has been used on a set of human CSF samples which included MS samples and shown to produce signals very similar to the SMI35-based assay [38]. A second generation pNF-H assay, using a novel monoclonal pNF-H capture antibody screened originally by ability to capture pNF-H from concentrated protein solutions, has also been described and is marketed by EnCor Biotechnology (M*α*-pNF-H ELISA) [39]. These ELISA methods have been used to measure pNF-H in serum in several neurological conditions including aneurysmal subarachnoid haemorrhage [40], amyotrophic lateral sclerosis [39], Leber's hereditary optic neuropathy [41], and neonatal hypoxic-ischemic encephalopathy [42, 43], but no publication has as yet described the use of this assay on plasma or serum samples from MS patients. 

We have shown, however, that the C*α*-pNF-H ELISA is a powerful serum marker of spinal cord axon loss in mice with experimental autoimmune encephalomyelitis (EAE), an experimental model of MS. In this model, serum pNF-H levels are associated with axonal loss (*r* = −0.80,  *P* < .001) and disability (*r* = 0.75,  *P* < .001), providing evidence that serum pNF-H levels accurately measure axon loss during inflammatory neurodegenerative changes in the CNS [44]. Interestingly, a drug known to ameliorate the EAE phenotype in these mice also greatly reduced the blood pNF-H levels. This suggests that serum pNF-H levels might be used in animal models to discover novel MS drugs and, if established in humans, to monitor the effectiveness of therapies in MS patients. In preliminary studies, we have also used the M*α*-pNF-H ELISA to show that serum pNF-H levels are elevated in around 10% of RRMS patients who show more rapidly progressing disease, suggesting that previously reported changes in CSF pNF-H levels in association with disease progression in MS could also be reflected in serum. Importantly, we have also been able to confirm the presence of NF-H peptides in these samples by MALDI-TOF mass spectrometry methodology, further validating the likely specificity of this ELISA methodology for detecting NF-H peptides [45].

## 6. Conclusion

The detection of NF subunits holds considerable promise as a means to monitor axonal loss in in MS patients. Work is being focused on the development of yet more specific and sensitive assays for NF proteins of utility both in CSF and blood. It will also be important to understand how these proteins are released from axons degenerating as a result of the MS disease process. While pNF-H released into the CSF of aneurysmal subarachnoid hemorrhage patients is mostly intact and unproteolyzed [40], we do not currently know what form is present in the blood of these patients, or in fact, any other group of patients. This raises the interesting possibility that NF subunits that are released with damage may be processed in disease-specific ways, and that these products might be detectable with refined assays of appropriate specificity. If this is correct, it may eventually be possible to detect axonal loss specifically due to the MS disease process. Future studies should therefore aim to develop more sensitive methods for measuring the various NF subunits in CSF, plasma, or serum to ascertain whether these biomarkers will be useful in the clinic for predicting MS onset, and for monitoring MS progression and response to therapy.

##  Disclosure

G. Shaw holds equity in EnCor Biotechnology Inc., a company that commercializes antibodies for some of the ELISA methods that are discussed in this paper, and may benefit by receiving royalties or equity growth.

## Figures and Tables

**Table 1:** Summary of studies assessing the utility of neurofilaments as biomarkers of axonal damage in patients with multiple sclerosis (MS) and clinically isolated syndrome (CIS).

Biomarker	Fluid	Study design	Observations	Associations with clinical measures	Ref.
NF-L	CSF	RRMS (*n* = 60); LP on trial initiation and 2 yr	78% patients showed ↑ at 0 or 2 yrs. Associated with recent relapse. No relation with age, gender, or disease duration.	EDSS 0 yr (*r* ^2^ = 0.27, *P* < .05); 2 yr (*r* ^2^ = 0.35, *P* < .01)	[16]

NF-L	CSF	RRMS (*n* = 41), SPMS (*n* = 25), healthy (*n* = 50)	↑ mean level in all MS subtypes. Highest during acute relapse.	EDSS (ns)	[17]

NF-L	CSF	CIS (*n* = 38), RRMS (*n* = 42), SPMS (*n* = 28), PPMS (*n* = 6); diagnostic LP	MS/CIS > controls. ↑ NF-L associated with relapse, T2 lesion number, Gd enhancing lesions. ↑ in CIS that convert to RRMS.	EDSS (*r* = 0.192, *P* < .05)	[18]
NF-L	CSF	RRMS (*n* = 16), SP/PP MS (*n* = 18)	MS > controls	EDSS (*r* = 0.41, *P* < .05)	[23]

NF-L	CSF	RRMS (*n* = 47)	MS > controls	—	[24]

NF-L	CSF	CDMS (*n* = 47)	MS > controls	—	[25]

NF-L	CSF	RRMS (*n* = 65), SPMS (*n* = 10), PPMS (*n* = 20); LP on initiation. Followup at 5 and 14 yrs.	↑ NF-L associated with recent relapse; associated with 3-fold ↑ in risk of developing high MSSS; more likely to convert from RRMS to SPMS.	MSSS 14 yr (*r* = 0.3, *P* < .01)	[26, 27]

NF-H	CSF	CIS (*n* = 38), RRMS (*n* = 92), SPMS (*n* = 28), PPMS (*n* = 6); diagnostic LP	MS > controls. SP/PPMS > RRMS. ↑ levels at CIS do not predict conversion to RRMS. ↑ with relapse. Correlated to age.	EDSS (*r* = 0.253, *P* < .01)	[18]

NF-H	CSF	RRMS (*n* = 11), SP/PPMS (*n* = 23); LP on trial initiation and 3 yr	SP/PPMS > RRMS.	EDSS 3 yr (ns trend)	[19]

NF-H	CSF	CIS (*n* = 52), RRMS (*n* = 38)	CIS > controls. ↑ acute relapse.	Correlated with EDSS for CIS and RRMS.	[28]

NF-H	Plasma	RRMS (*n* = 30)	Median levels RRMS > controls	—	[20]

Abbreviations: NF-L: neurofilament light; NF-H: neurofilament heavy; CSF: cerebrospinal fluid; RRMS: relapsing-remitting MS; SPMS: secondary progressive MS; PPMS: primary progressive MS; CDMS: clinically definite MS; LP: lumbar puncture; Gd: gadolinium; EDSS: expanded disability status scale; MSSS: multiple sclerosis severity scale; NS: nonsignificant; Ref: reference.
